# Imaging Transient Blood Vessel Fusion Events in Zebrafish by Correlative Volume Electron Microscopy

**DOI:** 10.1371/journal.pone.0007716

**Published:** 2009-11-06

**Authors:** Hannah E. J. Armer, Giovanni Mariggi, Ken M. Y. Png, Christel Genoud, Alexander G. Monteith, Andrew J. Bushby, Holger Gerhardt, Lucy M. Collinson

**Affiliations:** 1 Electron Microscopy Unit , London Research Institute, Cancer Research UK, London, United Kingdom; 2 Vascular Biology Laboratory, London Research Institute, Cancer Research UK, London, United Kingdom; 3 The Nanovision Centre, School of Engineering and Materials Science, Queen Mary University of London, London, United Kingdom; 4 Electron Microscopy Facility, Friedrich Miescher Institute, Basel, Switzerland; 5 Gatan UK, Abingdon, United Kingdom; Dartmouth College, United States of America

## Abstract

The study of biological processes has become increasingly reliant on obtaining high-resolution spatial and temporal data through imaging techniques. As researchers demand molecular resolution of cellular events in the context of whole organisms, correlation of non-invasive live-organism imaging with electron microscopy in complex three-dimensional samples becomes critical. The developing blood vessels of vertebrates form a highly complex network which cannot be imaged at high resolution using traditional methods. Here we show that the point of fusion between growing blood vessels of transgenic zebrafish, identified in live confocal microscopy, can subsequently be traced through the structure of the organism using Focused Ion Beam/Scanning Electron Microscopy (FIB/SEM) and Serial Block Face/Scanning Electron Microscopy (SBF/SEM). The resulting data give unprecedented microanatomical detail of the zebrafish and, for the first time, allow visualization of the ultrastructure of a time-limited biological event within the context of a whole organism.

## Introduction

Progress has been made in the field of high resolution electron microscopy to the point where structural determination of single molecules is becoming routine. However, the challenge of obtaining high resolution structural data from bulk samples of cells, tissues and whole organisms remains largely unexplored.

Transmission electron microscopy (TEM) overcomes the resolution limitation inherent in light microscopy but the requirement for ultrathin sections introduces sampling artefacts and masks interactions between cells and their environment in 3D space. This is a particular problem when dealing with complex, heterogenous samples including tissues and whole organisms. Where studies have been undertaken to map the ultrastructure of whole organisms, they have required experienced electron microscopists working over long time frames to collect, image and analyse thousands of serial sections, as in the mapping of neural networks in *Caenorrhabditis elegans* [1].

Scanning electron microscopes (SEMs) can image large samples but typically produce topographical or compositional information from only the surface layer. Recent innovations have combined SEM imaging with *in situ* sectioning, leading to a paradigm shift in high resolution volume imaging of biological samples. In Focused Ion Beam/Scanning Electron Microscopy (FIB/SEM) [2], [3], [4] and Serial Block Face/Scanning Electron Microscopy (SBF/SEM) [5], [6], [7] a slice of material is removed *in situ* and the revealed surface is imaged using the scanning electron beam. This cutting and imaging process is repeated sequentially to automatically collect a stack of high resolution images through the sample. In the FIB/SEM a gallium ion beam, which is inherently destructive, is used to ‘mill’ or ‘sputter’ away material whereas the SBF/SEM uses a modified ultramicrotome inside the SEM chamber to remove material using a diamond knife.

Although FIB/SEM and SBF/SEM give similar results, the mechanism of *in situ* sectioning means that the two techniques have distinct but complementary applications. Ion beam milling is currently limited by speed and surface area (to a maximum block face of ∼0.5 mm^2^) but can be targeted to specific areas, mill slice thicknesses down to ∼10 nm and cut through materials with different hardnesses in a single sample. In contrast, the diamond knife can cut surface areas in excess of 0.5 mm and the speed of cutting is high and independent of surface area, but section thickness is limited to ∼25 nm. Development of these techniques has been driven by neurobiology, where traditional EM cannot resolve the conflict between volume imaging of complex neural networks and high resolution imaging of individual neuronal connections [3], [5], [6]. Serial images have been collected through small volumes of brain tissue (∼300 µm^3^) with a lateral resolution of 4 nm^2^/pixel and an axial resolution of 40 nm [3].

We set out to develop and apply volume EM techniques to study another highly complex three-dimensional network, that of the developing circulatory system. The formation of new blood vessels, angiogenesis, is crucial in the patterning of the vascular system during vertebrate embryonic development in normal physiology and in pathological settings such as chronic inflammation, tumour progression and metastasis. During angiogenesis, endothelial cells (ECs) extend long filopodia to sense their environment and display a highly migratory behaviour [8] towards morphogen gradients [9]. Filopodia from two ECs must make contact and connect, an event known as anastomosis, giving rise to lumenized vessels able to carry blood flow. Anastomosis is a key step that has received little attention but is critical for the formation of new functional vessels and could yield novel specific targets for the inhibition of angiogenesis during tumour formation. Live confocal microscopy can be used to image anastomosis in the Tg(*fli1*: EGFP)*^y1^* [10] transgenic zebrafish embryo where ECs express green fluorescent protein (GFP). As with any fluorescence microscopy technique, information is limited to the distribution of the tagged protein and tells us nothing about the ultrastructure of the local microenvironment. However, traditional EM cannot resolve the conflict between volume imaging of the microanatomy of a large hierarchical branching blood vessel system and ultrastructural imaging of vessel formation in relation to surrounding cells and tissues *in vivo*.

We set out to develop a method combining live confocal and volume EM imaging of Tg(*fli1*: EGFP)*^y1^* [10] transgenic zebrafish embryos to map the temporal and spatial point of a transient biological event (blood vessel fusion) in relation to the surrounding tissue microenvironment. Live confocal imaging was used to follow endothelial cell growth to the point of contact between neighbouring EC sprouts. The targeted nature of FIB/SEM was critical in relocating the area of interest and mapping the microanatomy of the early sprouts through volumes of ∼50,000 µm^3^. SBF/SEM was then used to image the microanatomy of the post-fusion vessel in later stage embryos through volumes in excess of 120,000 µm^3^, where speed and surface area of sectioning become critical. The resulting large volume EM datasets give unprecedented ultrastructural detail of the zebrafish trunk microanatomy, encompassing not only the vasculature but also the epidermis, pigment cells, myotomes, notochord, neural tube and associated neurons. These techniques have enabled us to locate a transient event of 3 µm^3^ in a total volume of ∼12,600,000 µm^3^, and could be applied to any model organism, tissue or cell where small areas of interest need to be located within a large integrated network.

## Results

### Preliminary Studies of Anastomosis in Zebrafish

**Figure 1:**
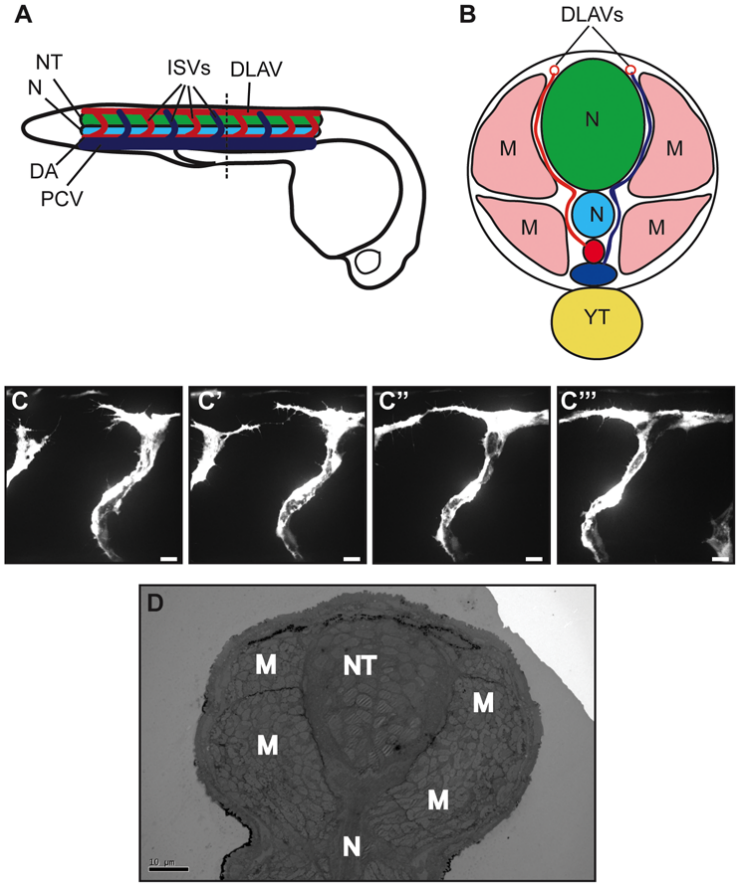
Preliminary studies of anastomosis in zebrafish. (A) Longitudinal diagram of 48 hpf zebrafish embryo, with neural tube (NT, green), notochord (N, turquoise), posterior cardinal vein (PCV, dark blue), dorsal aorta (DA, red), intersegmental vessels (ISVs, dark blue and red according to origin) and dorsal lateral anastomotic vessel (DLAV) shown in the trunk/tail region. (B) Cross section of zebrafish trunk; muscle blocks (M, pink) and yolk tube (YT, yellow) are also shown. (C) *In vivo* live confocal imaging of the anastomotic process showing two adjacent ISVs sprouting from the dorsal aorta and extending filopodia which eventually fuse to form the DLAV. (D) Ultrathin TEM sections give a general overview of the fish anatomy but it is not possible to identify the ISVs or DLAV as they do not have the lumen characteristic of blood vessels at this point. Bar (C–C′′′) 10 µm, (D) 10 µm.

The sprouting, migration and anastomosis of the inter-segmental vessels (ISVs) in zebrafish embryos leads to the formation of the dorsal lateral anastomotic vessels (DLAVs), giving rise to a patterned vascular system in the trunk (Fig. 1A). Using *in vivo* time lapse confocal imaging of ISV sprouting in *Tg(fli1*:EGFP*)^y1^* [10], one can observe the ISVs extending dorsally and branching rostrally and caudally upon reaching the dorsal aspect of the embryo [11] (Fig. 1C). This leads to contact between neighbouring ISVs and anastomosis (Fig. 1C and Movie S1 online). The result is the formation of two parallel, bilateral DLAVs running along the dorsal aspect of the embryo (Fig. 1B).

Electron microscopy was used to overcome the resolution limits of confocal imaging and obtain information about the interaction between ECs and the surrounding tissues. TEM of transverse ultrathin sections of a resin-embedded embryo revealed general ultrastructure of the trunk including the position of the notochord (N), neural tube (NT) and muscle blocks (M) with respect to the main axial blood vessels (Fig. 1D). This anatomical information was the basis for orientation and analysis of the sample in the FIB/SEM and SBF/SEM. However, it was extremely difficult to identify ISV endothelial sprouts or filopodia in ultrathin sections as they do not appear to have a characteristic lumen and travel through a volume of at least 50,000 µm^3^ (estimated from confocal data).

### Sample Preparation for FIB/SEM of Zebrafish

**Figure 2:**
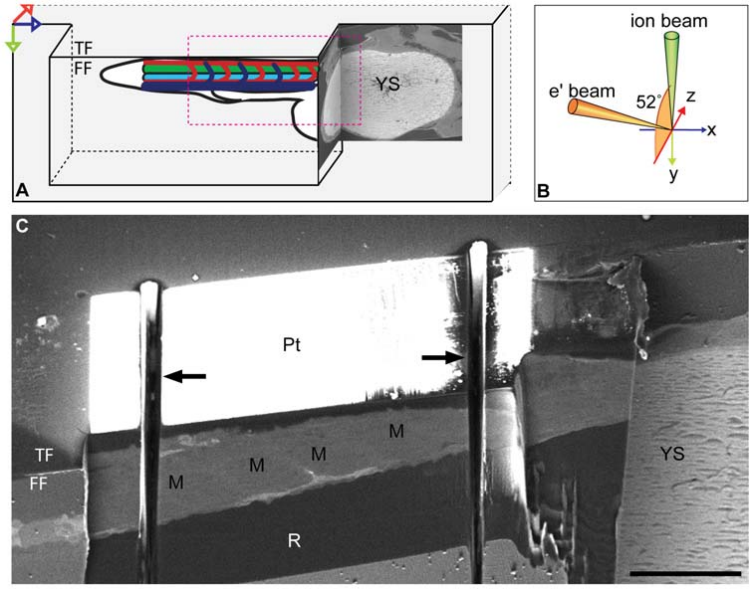
Sample preparation for FIB/SEM of zebrafish. (A) Diagram showing orientation of the zebrafish after resin embedding and trimming with a diamond knife. The AOI in the zebrafish tail is placed in an overhang of resin to optimise milling and mimise curtaining artefacts. The head and yolk sac (YS) are used as markers for locating the AOI, and are exposed during trimming, seen here in BSE images from the FIB/SEM. Top face (TF), front face (FF). Arrows represent x (blue), y (green) and z (red) axes. (B) Orientation of the electron and ion beams with respect to the front face (FF) of the block. (C) Platinum (Pt) was deposited onto the top face (TF) of the block to stabilise the edge during milling. The sample was coarse milled with the gallium ion beam to expose muscle blocks (M) at the predicted area of interest (AOI) prior to the milling run. Trenches (arrows) were then milled on either side of the AOI to reduce artifacts due to redeposition of sputtered material on the blockface. Resin (R). Bar (C) 100 µm.

Preliminary imaging of resin-embedded zebrafish in the FIB/SEM indicated that standard TEM embedding protocols using osmium and tannic acid produced suitable atomic number contrast for good backscattered electron (BSE) imaging. A mould for embedding FIB/SEM samples was designed to aid in orientation of the sample and trimming in the ultramicrotome, minimise redeposition of sputtered material back onto the block face by positioning the sample in an overhang of resin, and produce a block which is easy to mount in the SEM for correct orientation of the block face with respect to the ion and electron beams (Fig. 2A, B). Excess resin was trimmed away to allow viewing of the embedded embryo using transmitted light. The block was reorientated to align the trunk to the microtome knife-edge allowing further trimming to a smaller Area Of Interest (AOI). Part of the yolk sac was exposed on the recessed block face to aid relocation of the AOI in the FIB/SEM, platinum was deposited onto the top face of the block to protect the edge during milling and trenches were milled either side of the AOI to minimise redeposition of sputtered material back onto the block face (Fig. 2C). The face was milled at high ion current until the myotomes were exposed. The AOI could then be further narrowed from the position of the somite boundaries.

### Localisation of the ISVs by FIB/SEM

**Figure 3:**
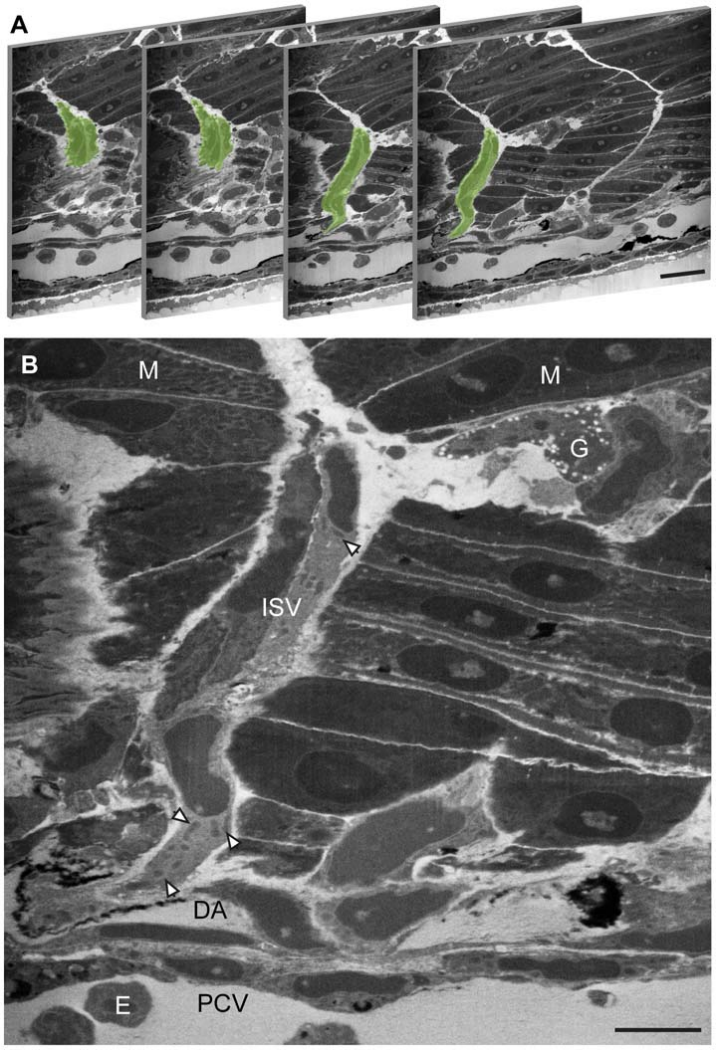
Localisation of the intersegmental vessels by FIB/SEM. (A) FIB/SEM sections of a 32 hpf zebrafish embryo trunk with the ISV highlighted in green. Slices are non-consecutive sections 661, 669, 687 and 691 where one slice is 50 nm thick. (B) The pericardinal vein (PCV) and dorsal aorta (DA) can be observed as lumenized vessels containing nucleated erythrocytes (E). The ISV sprouts from the DA and migrates in the space between the muscle block (M) and midline structures. Subcellular structure can be resolved including mitochondria (arrows) and dense granules (G) . Bar (A) 30 µm, (B) 10 µm.

A dataset of ∼3.33×10^5^ µm^3^ at 56×56×50 nm^3^ voxel resolution was collected from a 32 hours post fertilization (hpf) embryo (Movie S2 online). Localisation of the pericardinal vein (PCV) and dorsal aorta (DA) was straightforward as they have a characteristic lumen and are large enough to appear in many consecutive sections (Fig. 3). The axial vessels are formed by an EC monolayer displaying characteristic elongated nuclei and containing nucleated erythrocytes (E). An ISV is observed sprouting from the DA and migrating in the space between the somite and midline structures. Most muscle cells are observed to be multi-nucleated and the resolution of the data allows for the visualization of the z-discs in the muscle fibres and subcellular structures including dense granules (G) and mitochondria (M, arrowheads) (Fig. 3B). However, there are many different non-endothelial cells which occupy the inter-somitic space between the NT and the somite boundary which made it difficult to follow the ISVs through the volume and identify the point of anastomosis. It was therefore essential to develop a correlative light/volume EM strategy.

### Identification and 3D Reconstruction of the Anastomosing DLAV Imaged by Correlative Live Confocal - FIB/SEM

**Figure 4:**
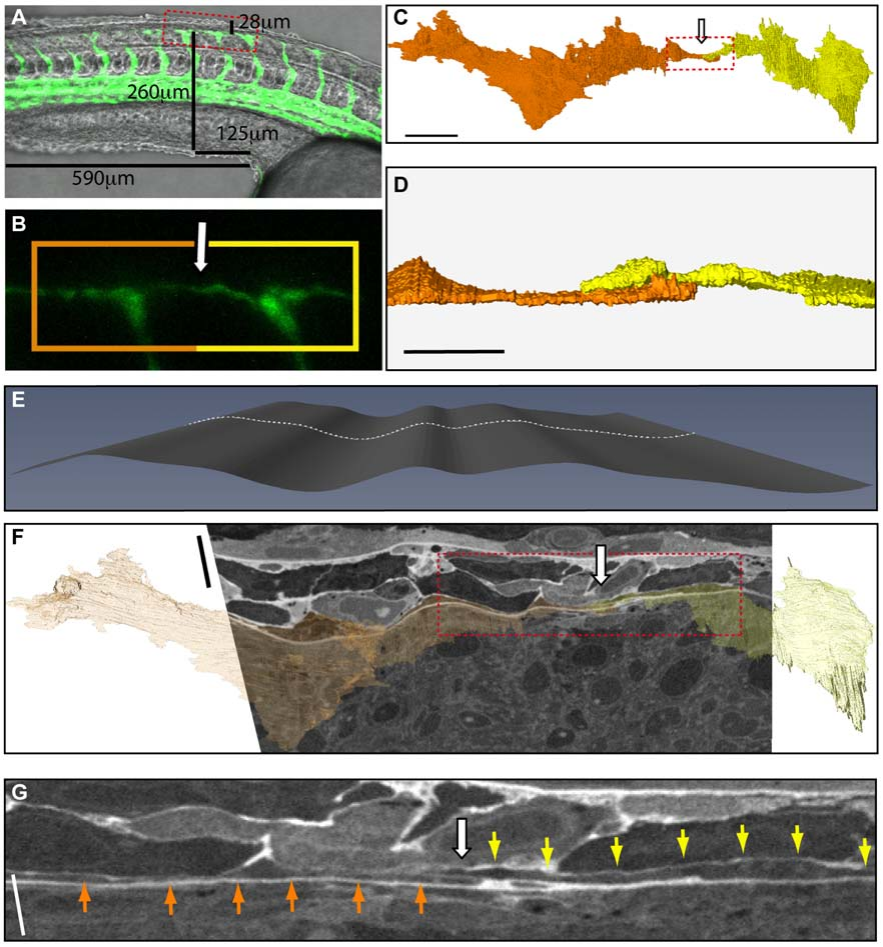
Anastomosis of the dorsal lateral anastomotic vessel. (A) Still from live confocal imaging of a 28 hpf Tg(*fli1*: EGFP)*^y1^* transgenic zebrafish at the point of anastomosis of filopodia from adjacent ISVs, overlaid on a phase image of the zebrafish. Measurements of the AOI are made in relation to gross physical markers like the yolk sac and yolk tube. (B) High magnification image of boxed area in (A) showing the point of anastomosis (arrow) and indicating vessels reconstructed in (C), (D) and (F) (orange and yellow box). (C, D) Features within the FIB/SEM dataset were segmented using Amira (Visage Imaging Inc.). Endothelial cells from adjacent ISVs (orange and yellow) form a thin layer between the neural tube and the somites. At the point of anastomosis filopodia from each cell overlap and contact (arrow). (E) It is only possible to visualise the ECs in the raw data by creating a curving plane through the volume using software (CurvedSlice, Amira). (F) Overview of the CurvedSlice data with the 3D reconstruction overlaid to highlight the filopodia of the ECs which narrow to <500 nm in places. When viewed in two dimensions the CurvedSlice distorts the data but captures the entire filopodia in one section demonstrating continuity. (G) High magnification of the AOI (box in (F)) from the CurvedSlice. Bar (C) 10 µm, (D) 3 µm, (F) 5 µm unidirectional, (G) 2.5 µm unidirectional.

In order to pinpoint anastomotic events, a 28 hpf Tg(*fli1*: EGFP)*^y1^* [10] zebrafish embryo was visualized by fluoresence microscopy until sprouts from adjacent ISVs could be seen to contact one another. The embryo was fixed and imaged by confocal microscopy. Measurements were taken on the resulting micrographs to help relocate the AOI after TEM processing and resin embedding (Fig. 4A, B). Shrinkage of the zebrafish during embedding was minimal, allowing relocation of the AOI with confidence and the block was trimmed in the ultramicrotome accordingly. The original measurements were then used in the FIB/SEM to identify the AOI for milling. A dataset of ∼2.42×10^6^ µm^3^ at 72 nm^3^ voxel resolution was collected. The images were aligned and the neural tube, notochord and tip cells were segmented using Amira software (Visual Imaging Inc., Fig. 4C, D, F and Movie S3).

The tip cells heading the sprouts were identified by their position and morphological features observed in confocal micrographs. 3D reconstruction showed sprouts to be flattened, in some cases to less than 500 nm, as expected due to the spatial restrictions between the NT and the somites. At the point of contact, the two ECs overlay one another (Fig. 4C, D, F, G white arrow), a process that has been previously noted during anastomosis [12]. 3D reconstruction of cells in close contact to the AOI revealed the presence of a granular cell wrapping around the point of contact between the sprouts (Movie S3, green cell in reconstruction). Similar granular cells were observed in the predicted region of an adjacent EC fusion point (data not shown) and close to the sprouting ISV (Fig. 3B).

It is essential to note that the EC filopodia cannot be imaged in one orthoslice as they narrow towards the point of anastomosis and meander through the volume. Similarly, it would be impossible to contain the filopodia within a single ultrathin section using traditional EM techniques. In fact, it is only possible to visualise the whole filopodia using a software algorithm such as a CurvedSlice (Amira, Fig. 4E) in which markers are distributed through the 3D scalar field of the raw data at points of interest to create a curving plane (Fig. 4E, F). However, some distortion of the data and loss in resolution may occur in displaying the resulting three dimensional curving plane as two dimensional image (Fig. 4G). It should be noted that scale bars on CurvedSlice images are unidirectional, due to the 3D curve plane appearing flattened in the 2D image.

### Ultrastructure of the DLAV in 72hpf Zebrafish Imaged by SBF/SEM

**Figure 5:**
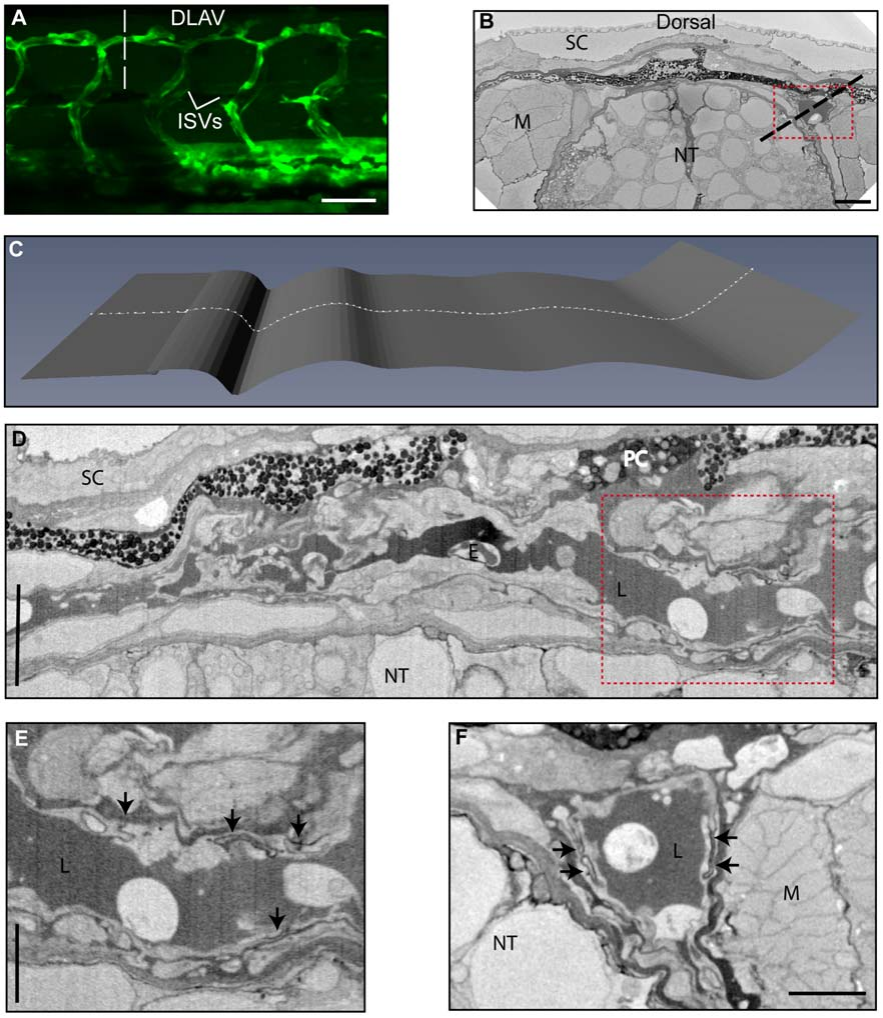
Ultrastructure of the formed dorsal lateral anastomotic vessel. (A) Confocal projecion of 72 hpf Tg(*fli1*: EGFP)*^y1^* transgenic zebrafish showing the fully formed DLAV. (B) Transverse section through 3View SBF/SEM dataset showing both DLAVs (dashed line in (A)). Scales (SC), muscle blocks (M), neural tube (NT). (C) Profile of the CurvedSlice (Amira) through the volume which selects one of the DLAVs (dashed line in (C)). (D) Overview of the CurvedSlice data showing the lumenized DLAV. Lumen (L), erythrocyte (E). (E) Higher resolution image of the DLAV from the boxed area in (D) showing electron dense patches between ECs which may represent cell-cell junctions (arrows). (F) Transverse section through the DLAV. Bar (A) 50 µm, (B) 5 µm, (D) 5 µm unidirectional, (E) 2.5 µm unidirectional, (F) 2.5 µm.

The ultrastructure of the formed DLAVs in 72 hpf embryos (Fig. 5A) was investigated using SBF/SEM (3View, Gatan Inc.) which is particularly suited to fast data collection from larger samples. A dataset of ∼1.2×10^5^ µm^3^ at 24×24×50 nm voxel resolution was collected (Fig. 5B–F and Movies S4, S5 and S6 online). The DLAVs are lumenized (L) by 72 hpf and erythrocytes (E) can be observed, as would be expected due to active circulation. The diameter of the DLAV varies considerably through the dataset and CurvedSlice projections (Amira) are required to visualise the entire vessel lumen (Fig. 5C–E). At this stage in development the DLAV is almost subcutaneous, with only pigmented cells separating the vessel from the dermis. Resolution is sufficient to see electron dense patches between ECs which are likely to represent cell-cell junctions (Fig. 5E, F, arrows). ECs lining the vessel lumen display typical elongated nuclei as for the axial vessels. Interestingly, there does not appear to be an open lumen within the ISVs, which correlates with observations in *Tg(fli1:eGFP; gata1:DsRed)* embryos, where blood flow can be imaged by confocal microscopy. In such embryos the flow in the ISVs appears infrequent and the erythrocytes seem to squeeze through the ISV, suggesting a lack of open lumen in the absence of flow (data not shown).

### Concept of High Resolution Anatomical Atlases from Volume EM Datasets

**Figure 6:**
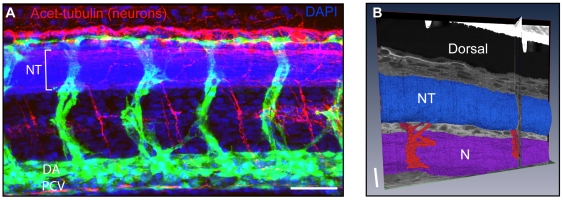
3D reconstruction of neuronal projections in a FIB/SEM dataset originally collected for a vascular study. (A) 48 hpf Tg(*fli1*: EGFP)*^y1^* transgenic zebrafish displaying formed ISVs and DLAV (green) which grow around and over the NT (blue, DAPI staining). The DLAV lies underneath bundles of neurofilaments (acteylated tubulin, red). Neuronal projections from the NT can also be observed. (B) 3D reconstruction and segmentation of a FIB/SEM dataset showing neurons (red) exiting the NT (blue). 3D rendered features are shown overlying x, y and z orthoslices from the original dataset. Notochord (N). Bar (A) 50 µm, (B) 10 µm.

To assess whether volume EM data could be used to identify other structures besides blood vessels, neuronal projections known to emanate from the neural tube were investigated. Immunofluorescence staining of 48 hpf zebrafish embryos for acetylated tubulin (neuronal marker) revealed neuronal projections from the neural tube into the space between the somite and the medial structures (Fig. 6A). Neuronal projections were identified in a FIB/SEM dataset by this positional information and by the characteristic dense content of neurofilaments (Fig. 6B and data not shown). Furthermore, data obtained from both FIB/SEM and SBF/SEM reveal vast quantities of information about tissue ultrastructure and organisation. Muscle fibres, z-discs and mitochondria are resolved (Fig. 3), neuronal synapses can be observed in the NT (data not shown) and structural detail of pigment cells and scales are evident (Fig. 5).

## Discussion

We have developed a method combining live fluorescence imaging with automated volume EM to produce unprecedented three-dimensional structural and functional detail of a transient biological process *in vivo*. This technique could find wide application in the fields of cell biology, vascular biology, developmental biology, pathology and neuroscience.

Currently, the main limitation of volume EM involves the pixel density and scan time during image collection in the SEM. When imaging at high resolution, the field of view is limited; conversely when imaging a large field of view, the resolution is limited. In order to locate a transient fusion event in a complex vascular network, a roadmap of the network microanatomy was created and followed to the area of interest using a large field of view but at some sacrifice to resolution. Future developments in microscopes, detectors and montaging of 3D image stacks will resolve this issue and allow us to image the subcellular details of anastomosis. In the meantime, knowledge of the ultrastructure of the ISVs, ECs and other cells in close contact to the fusion event should allow us to target the AOI more specifically, allowing us to minimise the field of view and therefore maximise the image resolution.

Additional challenges remain in minimising artifacts arising from the ion beam-material interaction during FIB/SEM milling [13], [14] and improvements in software, hardware and storage to handle datasets in excess of 10 GB. Interactive segmentation of data by hand is time-consuming and tedious and as yet there is no software for reliable automatic volume rendering, automatic segmentation of membranes or template matching [15], [16] in volume EM datasets. However, recent progress has been reported in feature tracking of axons through brain tissue in SBF/SEM datasets [17].

Morphological correlation of fluorescent markers has proved sufficient to relocate areas of interest within volumes, and could be applied in combination with recent advances in mapping large volumes in the light microscope. Reconstructions of zebrafish early embryonic development by scanned light sheet microscopy provide a ‘digital embryo’ with a database of cell positions, divisions and migratory tracks [18]. Volume light microscopy datasets could be overlayed onto the anatomical map provided by volume EM to allow structural identification of single cells *in vivo*.

However, correlative volume EM will reach it's full potential when markers for proteins of interest can be detected directly in the SEM forming a basis for understanding genetic and proteomic information *in vivo*. For example, electron-dense markers such as gold or ferromagnetic particles could be detected directly within tissues due to atomic number contrast. Applied to clinical biopsies, volume EM could be used to analyse delivery, distribution, uptake and efficacy of marker-conjugated therapeutic agents or antimicrobials in tumours or infected tissue respectively.

Amongst the many advantages of volume EM is the ability to automate large scale data collection. Indeed, the speed with which this data can be collected–as fast as 15 hours for 1,000 sections at 50×24×24 nm voxel resolution in the SBF/SEM–means that complete high resolution ultrastructural atlases of model organisms and tissues are within reach. For instance, different developmental stages of zebrafish, *Caenorhabditis elegans*, *Drosophila melanogaster*, *Schizosaccharomyces pombe* and *Saccharomyces cerevisiae* as well as standard cell lines could be mapped, to name but a few. Complete ultrastructure of wild type organisms could be compared to knockout and gene-silenced mutants to give clues to function from ultrastructural changes.

The datasets generated using these techniques for following one process also contain a wealth of information regarding other processes or functions of the organism. We have demonstrated that we can identify, follow and reconstruct neurons in a dataset originally collected to analyse blood vessel formation. We believe that making volume EM data available to the wider research community by the creation of an open access repository would maximise the potential of these techniques across multiple disciplines and revolutionise biological imaging.

## Methods

### Zebrafish and Immunofluorescence

The *Tg(fli1*:EGFP*)^y1^* transgenic line [10] was maintained in standard conditions as previously described [19]. For immunofluorescence staining, embryos were fixed in 4% paraformaldehyde (PFA) for 2 h at 4°C, blocked for 1 h at room temperature (RT) in 10% goat serum/2% BSA/0.5% Triton in PBS, incubated overnight at 4°C with anti-acetylated tubulin antibody (T7451, Sigma) followed by incubation overnight at 4°C in secondary antibody.

### TEM

Zebrafish were fixed in 2% PFA/1.5% glutaraldehyde in 0.1 M sodium cacodylate (pH 7.4) for 1 h, post-fixed in 1% osmium tetroxide/1.5% potassium ferrocyanide for 1 h and stained with 1% tannic acid in 0.05 M sodium cacodylate (pH 7.4) for 45 min at RT. Samples were dehydrated through an ethanol series, transferred to acetone and embedded in Durcupan resin according to the manufacturer's instructions (TAAB Laboratories Equipment Ltd). Ultrathin sections of 80 nm were cut, post-stained with lead citrate and viewed in a Tecnai Spirit Biotwin 120 keV TEM (FEI Company). Images were captured using Ultrascan and Orius CCDs (Gatan Inc.) with TIA (Tecnai Imaging and Analysis, FEI Company) and Digital Micrograph software (Gatan Inc.) respectively.

### Focused Ion Beam/Scanning Electron Microscopy (FIB/SEM)

Zebrafish were embedded as described in ‘TEM’ above. The blockface was trimmed with a razor blade to reveal either a transverse or a lateral section through the trunk of the fish, and polished in a UCT ultramicrotome (Leica Microsystems UK) using a 90° diamond trimming knife (Diatome) so that all faces were smooth and perpendicular. Excess resin was trimmed away from the top and back surfaces of the block to reduce the volume of non-conductive material and the sample was mounted onto a 12.5 mm aluminium stub (Agar Scientific) using silver paint (Agar Scientific) and carbon-coated (Blazers CED030) to reduce charging. The sample was inserted into the stage of a Quanta 3D FEG FIB/SEM (FEI Company), raised to a working distance of 10 mm (the coincidence point of the electron and ion beams) and tilted to 52°. The block was imaged at an accelerating voltage of 5 keV and a current of 1.28 nA. The position and orientation of the zebrafish within the block was determined using secondary electron imaging mode (SEI, Everhardt Thornely Detector), whereas atomic number contrast of osmium within the tissue was imaged using a solid-state backscattered electron (BSE) detector [5], [3]. An area of the blockface up to 80,000 µm^2^ was coarse-milled at an ion beam current of 65 nA (accelerating voltage 30 keV) to remove excess resin until tissue could be seen. Subsequently a 1 µm layer of platinum was deposited using the chamber gas injection system on the block face above the AOI to stabilise the edge during milling [3], [13] and trenches were milled on either side of the AOI to minimise redeposition of sputtered material back onto the blockface. The blockface was polished stepwise at 30, 15, 7, 5 and 3 nA until the surface was smooth. Milling conditions for the Slice and View run (S&V|, FEI Company) were 130×10 µm at 50 nm slice thickness at a current of 5 nA which gave a mill time of 73 s/slice. BSE imaging conditions were set at 5 keV accelerating voltage and 1.28 nA beam current with a dwell time of 30 µs, giving an acquistion time of 108 s/image. The image width was 114.5 µm at 2048×1768 pixels giving a final lateral resolution of 56 nm^2^/pixel. 693 images were collected over ∼35 h.

### Correlative Live-Confocal FIB/SEM

Embryos at 28 hpf were anaesthetized in systems water containing tricane (0.016%, pH 7) and immobilized in 0.2% agarose on glass bottom culture dishes (MatTek Corp.). Embryos were kept at 28.0°C in an environmental chamber. Time-lapse microscopy was carried out on a Zeiss Axiovert 200 M fitted with an LSM 5 Pascal system. EGFP was excited with 488 nm laser emission supplied by an Argon laser. Stacks were composed of several optical slices with 1 µm slice spacing, acquired every 2 mins. The time-lapse video is a 2D representation of projected stacks. Zebrafish were fixed in 2% PFA in PBS as soon as sprouts from the ISVs had contacted and the AOI containing the anastomosing blood vessels was imaged at 10× and 40× for correlative measurements. Zebrafish were processed as described previously and embedded in moulds designed to simplify the trimming and milling steps for FIB/SEM (TAAB Laboratories Equipment Ltd). Using measurements collected in the confocal and dissecting microscopes pre- and post-embedding, the zebrafish was manually trimmed and polished to a blockface of approximately 700 µm wide by 300 µm deep (including part of the yolk sac as an orientation marker) to expose a lateral cross section of the AOI at the blockface. This was mounted on an SEM stub and imaged in the FIB/SEM as described above. The blockface was coarse milled until the myotomes were revealed and the exact orientation of the fish established. Milling conditions for the S&V|run were 400×5 µm at 72 nm slice thickness at a current of 7 nA which gave a mill time of 69 s/slice. BSE imaging conditions were set at 5 keV accelerating voltage and 1.3 nA beam current with a dwell time of 30 µs, giving an acquistion time of 108 s/image. The image width was 148.6 µm at 2048×1768 pixels giving a final resolution of 72 nm^3^/voxel. 1778 images were collected over ∼87 h.

### Serial Block Face/Scanning Electron Microscopy (SBF/SEM)

Embryos at 72 hpf were imaged, fixed, embedded and trimmed as detailed above. The sample was trimmed to expose a transverse section through the trunk and inserted into the 3View microtome (Gatan Inc.) in the chamber of a Quanta 200 VP-FEG (FEI Company) with the block face aligned with the pole piece. In order to perform serial SEM of the block face [5], a 50 nm slice was cut from the face with a diamond knife and the freshly cut surface of the block was scanned, and this process was repeated sequentially to collect 1000 slices over ∼15 h. Imaging was performed at an accelerating voltage of 4 keV in low vacuum mode (0.3 Torr) at 2048×2048 pixels with a pixel resolution of 24 nm^2^.

### 3D Reconstruction and Segmentation of Data

FIB/SEM datasets were batch processed in Adobe Photoshop for optimal brightness and contrast, followed by alignment and reconstruction into a 3D volume using Amira (Visage Imaging Inc.). SBF/SEM datasets were aligned and reconstructed into a 3D volume using Digital Micrograph (Gatan Inc.) and Imaris (Bitplane Scientific Solutions). Features were segmented using Amira and movies were made in Amira and Imaris.

## Supporting Information

Movie S1 Live fluorescence imaging of blood vessel fusion in the Tg(fli1: EGFP)y1 transgenic zebrafish embryo, stills from which are shown in Fig. 1C.
(1.68 MB MOV)

Movie S2 x, y and z orthoslices from the aligned FIB/SEM dataset in Fig. 3 showing the main axial vessels of a 32 hpf zebrafish embryo (movie created in Amira software).
(3.41 MB MOV)

Movie S3 3D reconstruction of features from the correlative FIB/SEM dataset in Fig. 4. The neural tube (dark blue), notochord (mid blue), endothelial cells (orange and yellow) and cells in close contact to the point of anastomosis (green, purple, lilac) are shown. Movie created using Amira software.
(4.12 MB MOV)

Movie S4 x, y and z orthoslices from the 3View dataset of a 72 hpf zebrafish embryo in Fig. 5 (movie created in Imaris software).
(1.76 MB MOV)

Movie S5 3D volume of the SBF/SEM dataset in Movie S4 showing a Clipping Plane which ends in the AOI highlighting the dorsal lateral anastomotic vessels (movie created in Imaris software).
(2.53 MB MOV)

Movie S6 Higher resolution x, y and z orthoslices of the SBF/SEM dataset in Movie S4 focusing on the dorsal lateral anastomotic vessel in Fig. 5 (movie created in Imaris software).
(2.50 MB MOV)
